# Supplementary material for: Distributed recurrent neural forward models with synaptic adaptation and CPG-based control for complex behaviors of walking robots
Source: Front Neurorobot. 2015 Sep 25;9:10. doi: 10.3389/fnbot.2015.00010 (PMC4585172; doi:10.3389/fnbot.2015.00010)
Supplement: Supplementary file 7 [file DataSheet1.PDF]

Frontiers in Neurorobotics

Research Article
11 September 2015

Supplementary Material: Distributed Recurrent Neural Forward Models with Synaptic Adaptation and CPG-based control for Complex Behaviors of Walking Robots

Sakyasingha Dasgupta [1,2,4*], Dennis Goldschmidt [2], Florentin Wörgötter [1,2] and Poramate Manoonpong [2,3]

[1] *Institute for Physics - Biophysics, George-August-University, Göttingen, Germany*
[2] *Bernstein Center for Computational Neuroscience, George-August-University, Göttingen, Germany*
[3] *Center for Biorobotics, Maersk Mc-Kinney Moller Institute, University of Southern Denmark, Odense, Denmark*
[4] *Riken Brain Science Institute, 2-1 Hirosawa, Wako, Saitama, Japan*

Correspondence*:
Sakyasingha Dasgupta
Riken Brain Science Institute, 2-1 Hirosawa, Wako, Saitama, Japan,
sakyasingha.dasgupta@riken.jp

Neural plasticity for rich and uncertain robotic information streams

1 SUPPLEMENTARY TABLES AND FIGURES

Supplementary Table 1. Self-adaptive Recurrent Neural Network (reservoir) forward model parameters

Parameters	Values
Recurrent Network Size - N	30
Number of output neurons	3 (walking gaits)
Number of input neurons	1 (CTr motor signal)
Time step - Δt	0.037
Neuron time constant initialization - τ	10 ms
δ_c	10^-3
Scaling parameter - g	0.95
Connection probability - p_c	0.2
Neuron nonlinearity shape initialization - a_i	1.0
Neuron nonlinearity scale initialization - b_i	0.0
Neuron axillary bias- B_i	$N(0, 0.01)$
Input weight initialization - W^{in}	$U[-0.1, 0.1]$
Recurrent weight initialization - W^{rec}	$N\left(0, \frac{g^2}{\sqrt{p_c N}}\right)$

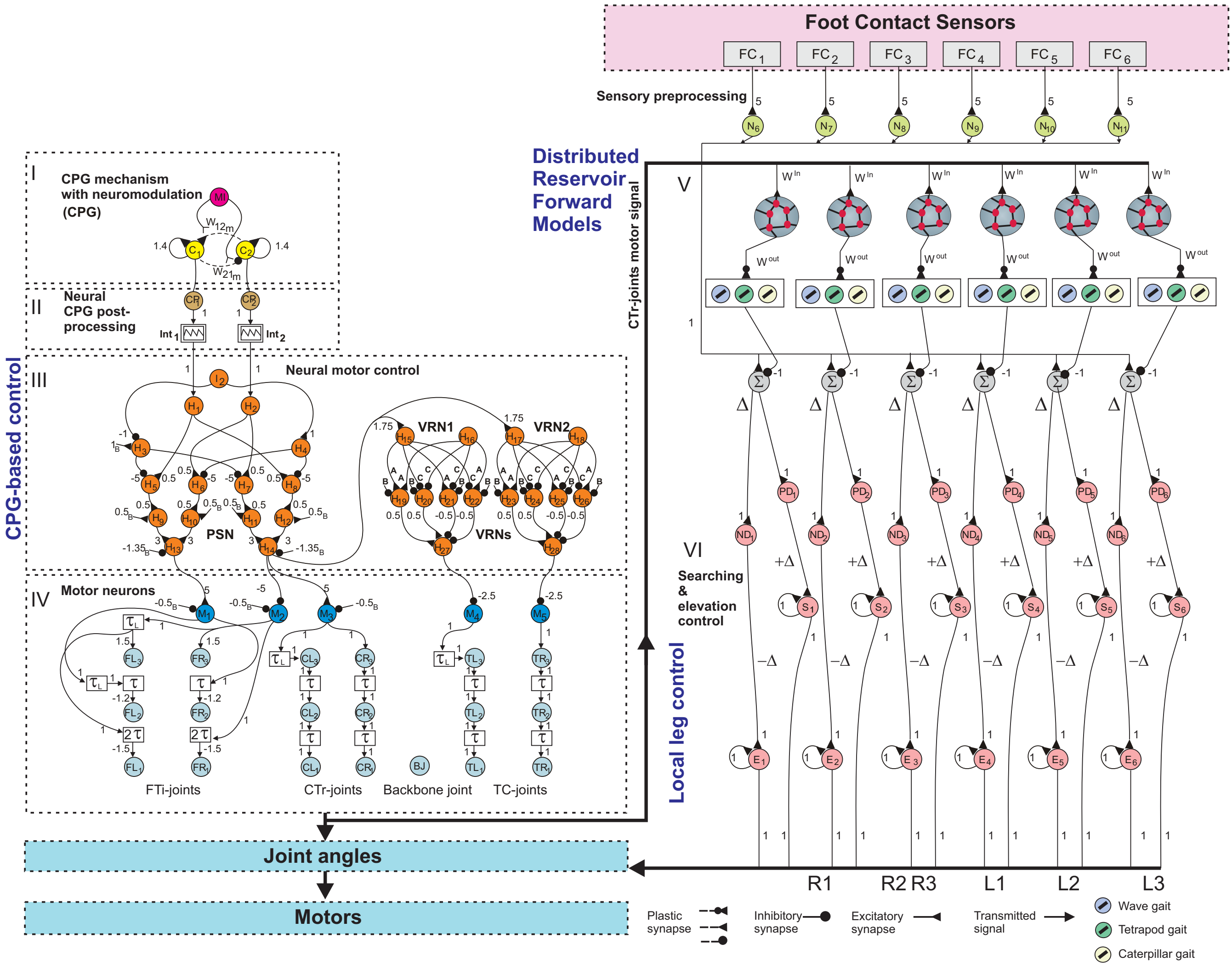

Figure 1. Main wiring diagram of the central pattern generator based control, the reservoir forward models and local leg controls. Single CPG-based control applied to AMOS II for locomotn. CPGs outputs are projected to PCPG (CPG post processing unit) which translate them into ascending and descending slopes, then these signals are fed to the PSN (phase shift network) component. The outputs of the PSN are projected to the F(R,L) and C(R,L) motor neurons (i.e. the FTi and CTr joints of the robot) through delay lines, as well as to the VRN (velocity regulating network). The VRNs outputs are projected to the T(R,L) motor neurons (TC joints) through delay lines. The CTr joint signals were then used as efference copies that feed as time varying inputs to each of the six reservoir forward models. This in turn is connected to the local searching and elevation controls.

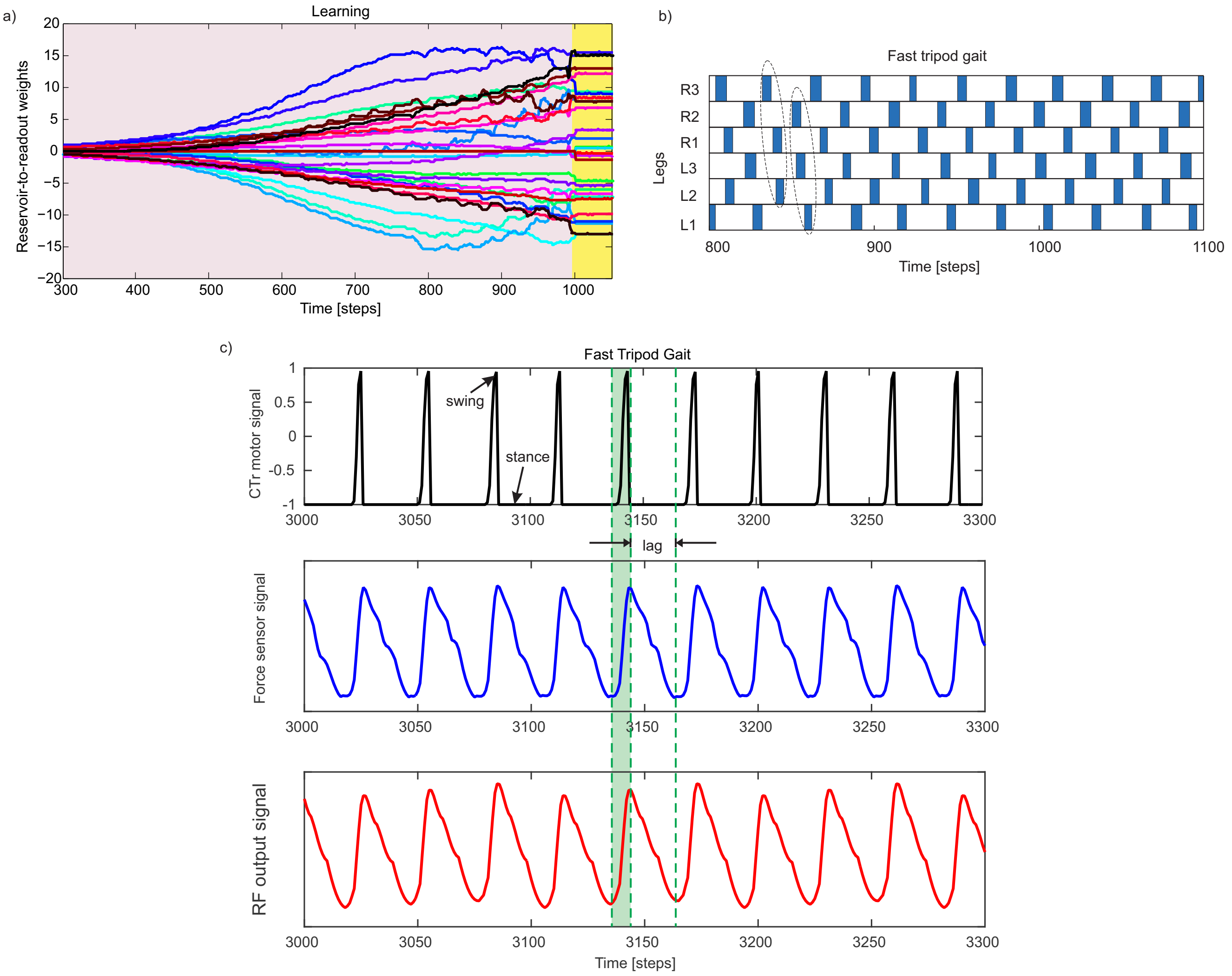

Figure 2. Learning of the reservoir forward model for tripod walking gait (a) Changes of 30 weights projecting to the first readout neuron (z_1) of the forward model of the right front leg (R_1) while walking with a fast tripod gait. (b) The gait diagram for the tripod gait. They are observed from the motor signals of the CTr-joints. White areas indicate ground contact or stance phase and blue areas refer to no ground contact during swing phase. As frequency increases, some legs step in pairs (dashed enclosures). (c) Top - The CTr-joint motor signal of the right front leg (R_1) for the tripod gait. This motor signal provides the efference copy or the input to the reservoir forward models, middle - the actual foot contact signal (force sensor signal under normal walking conditions) used as the target signal of the reservoir models, bottom - the predicted foot contact signal or the final learned output of the forward model for each walking gait (RF output signal). The green shaded region indicates the time interval between swing and stance phase for the CTr motor signal for the tripod gait.
